# Can Afro-Communitarianism Be Useful in Combating the Challenge of Human Interaction Posed by the COVID-19 Pandemic?

**DOI:** 10.3390/ijerph192114255

**Published:** 2022-10-31

**Authors:** Jonathan O. Chimakonam, L. Uchenna Ogbonnaya

**Affiliations:** 1 Department of Philosophy, University of Pretoria, Pretoria 0002, South Africa; 2 The Conversational School of Philosophy, University of Calabar, Calabar 540271, Nigeria

**Keywords:** Afro-communitarianism, human interaction, COVID-19, pandemic

## Abstract

Can Afro-communitarianism serve as a viable ideology for addressing the human interaction challenge posed by the COVID-19 pandemic? The ongoing pandemic poses many challenges to the normal functioning of societies around the world. For example, it has caused problems ranging from social, economic, and political disruption to various forms of hardship, including pain, suffering, and millions of deaths. One problem that is not attracting sufficient attention is a disruption to human interaction that leads to isolation, depression, mental health, and emotional crises. This paper will investigate whether Afro-communitarianism can function as an ideological option for addressing this challenge. This ideology, in our opinion, can foster social integration and the type of informal solidarity that engenders emotionally helpful interactions among humans. We will also argue that Afro-communitarian orientation can overturn the individualistic tendencies that hamper efforts aimed at curtailing the spread of the coronavirus.

## 1. Introduction

Communitarianism and individualism are two contesting ideologies when it comes to human relations. While communitarianism places communal values before those of the individual, individualism holds that individual endowments precede those of the community. Although these ideologies are global (in that they are operational in every cultural and geographic area), some African scholars argue that communitarianism is African in origin and that it is an ideology that sustained pristine Africa [1,2]. Their point is that while communitarianism is African, individualism is un-African.

Conversely, J. Obi Oguejiofor contends that communitarianism is not restrictively African. For him, we can understand communitarianism from three perspectives: (1) communitarianism is African, but not to the exclusion of non-African cultures and peoples; (2) communitarianism is African to the exclusion of non-African cultures and peoples; and (3) communitarianism is essentially African, given that it is an ideal that distinguishes African from non-African cultures and peoples [3]. While the first perspective is inclusivist, the second and third perspectives are exclusivist. Oguejiofor asserts that communitarianism is not exclusively and essentially African. Rather, he argues that non-Africans also practice this idea and that it is only from the first perspective that communitarianism can be said to be African.

Scholars such as Dismas Masolo [4], Munamato Chemhuru [5], etc., share the above view. The authors also share the understanding that there is a form of communitarianism that can be described as African, especially the version that is African culture-inspired, with the traits of solidarity, sharing, caring, mutual interdependence, and complementarity. Therefore, what is commonly known as Afro-communitarianism can be seen as a short form of African communitarianism (that is, an African culture-inspired type of communitarianism). Afro-communitarianism, as with the communitarianism discussed in Western scholarship, is a social organization principle that guides human conduct and interaction in society. This doctrine prioritizes the ideas of a relationship of solidarity that promotes mutual sharing, caring, interdependence, and complementarity. Masolo argues that it is “a doctrine or school of thought in social and moral philosophy” [4]. In this way, it is an ideology with pragmatic relevance. The relevance of Afro-communitarianism can be attested to by the extensive works written by certain African philosophers and scholars, such as Ifeanyi Menkiti [6], Jonathan Chimakonam [7], Chimakonam and Victor Nweke [8], Bernard Matolino [9]. These scholars have employed Afro-communitarianism to address certain burning issues, such as personhood, democracy, human dignity, rights, etc., confronting contemporary Africa and the entire human race.

Olúf${ẹ}^{´}$mi Táíwò holds that there are four primary forms of communitarianism (in this context, Afro-communitarianism): the methodological, the ontological, the axiological, and the epistemological [10]. Methodological communitarianism is an explanatory model employed to ground human relations in the community. Ontological communitarianism focuses on how all beings are communal and relational. This ontological framework explains how humans ought to relate to one another, just as all beings live communally. Epistemological communitarianism deals with collective knowledge and wisdom, which rest on the elders’ shoulders: these elders are the custodians of the community’s wisdom. Axiological communitarianism is concerned with the communal values that guide human behavior in society. Therefore, it dictates how humans ought to conduct themselves in a community, so that society remains orderly.

This paper will employ methodological and axiological communitarianism to address the challenges of human social interaction brought about by the COVID-19 pandemic. The COVID-19 disease is one of the deadliest and most feared diseases in human history, caused by a virus known as SARS-CoV-2, or the coronavirus. This disease has defied medical practice as there is yet to be a cure or credible preventive action to curtail its rapid spread. On this note, the World Health Organization (WHO) has prescribed certain measures, such as self-isolation, physical distancing, lockdown, etc., to reduce virus outbreaks. Even with these measures, the virus is still causing havoc; it has claimed millions of lives, increased poverty, destroyed social cohesion, and led to fear, hardship, uncertainty, etc. The COVID-19 pandemic has brought about the challenge of human interaction. To address this challenge, we intend to employ Afro-communitarianism (communitarianism, as articulated, inspired by African cultures). Our central thought is to demonstrate how Afro-communitarianism can be useful in addressing the challenge facing human interaction in this era of COVID-19.

We begin the paper with this introduction, which is immediately followed by a section on the crux of Afro-communitarianism. Thereafter, we will look at the COVID-19 pandemic and its impact on human interaction. This will be followed by a section on how Afro-communitarianism can be useful in addressing the challenge of human interaction posed by the COVID-19 pandemic. Finally, we shall conclude the paper by indicating some promising ideas that Afro-communitarianism harbors regarding human interaction in this COVID-19 era and future times.

### 1.1. The Crux of Afro-Communitarianism

We are not unaware that some Western scholars, such as Charles Taylor, Amitai Etzioni, Alasdair MacIntyre, etc., discuss ideas that are described as communitarianism in the Western intellectual sphere. For the purposes of this paper, we are not interested in engaging with their position or in comparing it with the version discussed in the literature on African philosophy. Here, we want to focus on Afro-communitarianism, and if, in the process, we discuss ideas that cut across both the African and Western philosophical traditions, it would simply show that there are common traits in both traditions. Afro-communitarianism, as a concept, consists of two words, namely, “Afro” and “communitarianism”. The word “Afro” is an abridged version of “African”. Afro-communitarianism is an ideology that highlights the relationship between the individual and their community in a typical African society. It holds that communal values come before individual endowments. As a result, individual identity or personality is believed to be shaped by the community, leaving little room for individual endowments such as rights, autonomy, freedom, etc. Because of this, many African scholars see this as the antithesis of individualism [4]. Hence, some scholars mistakenly argue that Afro-communitarianism is against individual interests.

On the contrary, Afro-communitarianism not only takes into account the community’s well-being but also recognizes individual interests. Afro-communitarianism entails that every individual’s interest within the community ought to be given adequate attention and be taken care of without reservation. The point is that community interest and well-being are taken seriously when the needs of the individuals who make up that community are taken seriously. That is to say that the individuals’ interest in the community is also the community’s interest since it represents a collection of individuals. This is one way of looking at Afro-communitarianism. In this way, Pantaleon Iroegbu [11] argues that the African version of communitarianism is one in which the community subsumes rather than consumes the individual.

Another idea that Afro-communitarianism brings to the table is the collective or communal path to addressing the needs of individuals in the community. Given that individuals exist in the community, one individual’s problem is the whole community’s problem. Therefore, in addressing this problem of the individual, we address the problem of the community. This makes problem-solving a collective effort and the duty of the community. The idea is that individuals do not exist alone. They exist with one another and are interconnected and interrelated, such that they become interdependent. If this is the case, as it is, then Afro-communitarianism entails that individuals in the community live by cooperating, sharing, and living in solidarity with one another because of their insufficient and interdependent nature. A. Wade Boykin succinctly elaborates this as he asserts that Afro-communitarianism

denotes an awareness of the interdependence of people. One’s orientation is social, rather than being directed toward objects. One acts in accordance with the notion that duty to one’s social group is more important than individual privileges and rights. Sharing is promoted because it signifies the affirmation of social interconnectedness; self-centeredness and individual greed are disdained [12].

The above assertion has some inherent elements that constitute and define Afro-communitarianism. They include: “(1) primacy of social existence, (2) sanctity of social bonds and relations, (3) transcendence of group deities and responsibilities over individual concerns, (4) anchoring of individual identity in the group, and (5) an emphasis on sharing and contributing in support of the group” [13].

Afro-communitarianism, as discussed above, highlights the fact that social bonds, relationships, and identity lead to collaborative care and responsibility toward society and the members of that society. In light of this, communal life entails living, feeling, and working together for the common good of all [14]. It is only in this sense that we can say it is opposed to individualism, following the doctrine of promoting mainly the welfare of the individual at the detriment of the community and other members of the community or of a value system that holds that “the individual is paramount and the community is a consequent” [14]. Some have pointed out that in Afro-communitarianism, the well-being of the collective (group) or community is emphasized above that of an individual [5,6,9], although they disagree as to whether it is the correct approach or not. Those who think it is the correct approach argue that an individual employs their wealth to promote the well-being of the community and other community members, by providing for them according to their needs. This act demonstrates that Afro-communitarianism promotes altruism and almsgiving [4]. It is believed that this social idea defined pristine Africa. Hence, we have the notion of Afro-communitarianism.

Afro-communitarianism could be said to imply an African version of communitarianism. If we agree with this definition, we are saying that the idea of communal living in Africa is distinct from the approach of those who are non-African. This is not actually the case. Communal living in Africa is, in so many ways, related to those methods found and practiced in non-African societies. However, communal living in Africa has certain African cultural nuances that make it African and not non-African. On this note, we hold that Afro-communitarianism is an African culture-inspired communal living model that stresses shared, mutual, and common goals, goods, interests, and values above the individual goal, right, interest, and value [15]. It is an ideal and practice in the African scenario that is communocentric or sociocentric and not egocentric. In this model, there is an orientation of mutual interdependence and complementarity. Hence, Afro-communitarianism is communal and complementary since the ideas of community, relationship, togetherness, co-existence, co-dependence, and co-responsibility toward the community and others are paramount [16]. Innocent Asouzu emphasizes this point when he contends that for the traditional Igbo society, “Without complementarity, human life would be unbearable, isolated struggles that easily lead to self-abandonment. We can now understand why the traditional Igbo never cease to sound the clarion call to complementarity and solidarity in times of crisis” [16]. This renders Afro-communitarianism an African value system that is community-oriented, in that people live as brothers and sisters and jointly strive to address the varied challenges or problems of life. Thus, community, togetherness, and communal existence, in Afro-communitarianism, are prized above individualism, but individual existence is taken seriously since the community does not exist without the individual. In all, Afro-communitarianism, by being communal, emphasizes egalitarianism, wherein all are treated with equality and individuals treat one another as brothers and sisters.

The notion of Afro-communitarianism is encapsulated in some African philosophical theories, such as Leopold Senghor’s Negritude [17]—the “self” relating with the “other”; Julius Nyerere’s *Ujamaa* [18]—humans relating to each other as members of a family (an extended family); Pantaleon Iroegbu’s Uwa ontology [19]—to be is to belong to each other, in *Uwa*; the Southern African notion of Ubuntu [20]—a person is a person through other persons; Innocent Asouzu’s *Ibuanyidanda* ontology [16,21]—to be is not to be alone or to be is to be in a mutually complementary relationship, being the missing link; Chris Ijiomah’s *Harmonious Monism* [22]—complements or contradicts coexisting as one; Thaddeus Metz’s *Relational Ethics* [23]—humans ought to coexist in the community so that their relationship affirms their identity and brings about solidarity (harmonious co-existence); Godfrey Ozumba and Chimakonam’s *Njikoka Amaka* [24]—to integrate is better than to be alone; Chimakonam’s *Ezumezu* [25,26,27]—to be made complete through sub-contraries integrating with, and complementing, each other within a whole, etc. These theories demonstrate that Afro-communitarianism promotes co-existence, complementarity, and solidarity as values that should be imbibed and practiced.

From the preceding discussions, we have focused on the methodological dimension of Afro-communitarianism. It is also pertinent to consider the axiological dimension of Afro-communitarianism. The central question is, what are the values that Afro-communitarianism promotes? These values, for example, can be deduced from the four principles that Asouzu provided: the *principle of integration*, which states that “anything that exists serves as a missing link of reality”; the *principle of progressive transformation*, which states that “all human actions are geared toward the joy of being”; the *ibuanyidanda imperative*, which states: “Allow the limitations of being to be the cause of your joy”; and the *truth and authenticity criterion*, which states: “Never elevate any world immanent missing link to an absolute instance” [21]. These four principles prescribe the necessity of interdependent and complementary interaction and the benefits of relating in such a mutual way within a community.

The type of mutuality prescribed by the principles above connotes that social harmony is the greatest good. Thus, Afro-communitarianism promotes the need to avoid anything that subverts or undermines this desired good. The *truth and authenticity criterion* specifically proscribes any act that negates equality, mutual co-existence, and interdependence. Such conduct is said to decrease life and human flourishing [23].

From the above, we can say that Afro-communitarianism holds that human actions and decisions must lead to harmony, life, and the common good of society since people live in a community. Gyekye summarizes this as he argues that society is moral if individuals demonstrate concern for others’ interests and needs. This deduction from the Afro-communitarian moral theory holds that “the community, through a harmonious relationship, must work for the fundamental human good of all and each individual must live in harmony and share in the fate of the other” [28]. A central good in the Afro-communitarian dynamics is human interaction, without which the idea of such a mutualizing ideology might be lost.

### 1.2. The COVID-19 Pandemic and Its Impact on Human Interaction

COVID-19 is a respiratory disease that affects the respiratory organs and tract. It is caused by the novel coronavirus (i.e., SARS-CoV2). The effect of this disease varies from person to person, depending on age, health conditions, and time of exposure of the individual to the virus. The symptoms range from mild to severe. These symptoms include body and muscle aches and pains, chills or fever, cough, dyspnoea (breathing difficulty or shortness of breath), headache, diarrhea and vomiting, loss of smell and taste, runny nose, sore throat, etc. [29]. The signs and symptoms of these diseases manifest between 2 and 14 days after infection. Although (medical) scientists have recently created several vaccines for prophylactic purposes, these vaccines’ potency is still in doubt, given that none has proven to be one hundred percent (100%) effective. A cure is yet to be found for the COVID-19 disease, which has claimed millions of lives and is still ravaging the entire globe.

Since “there is yet no effective treatment to provide immunity against infection and prevent the disease” [30], “COVID-19 might be amongst us for a while” [30]. Premised on this view, the governments of various nations of the world have put forward several measures/strategies recommended by the World Health Organization (WHO) to curtail the spread and transmission of the disease. They include isolation, physical distancing, the avoidance of handshakes and embraces, global and local lockdown, shutdown, wearing masks, washing hands thoroughly with soap and water, sanitizing hands and concrete surfaces, etc.

The persistence of COVID-19, with its many mutations and the above-stated measures to curtail its spread, may mean that individuals are being denied some of their fundamental human rights. Some of the rights affected include the right to life, the right to education, the right to equality and non-discrimination, the right to freedom of movement and liberty, the right to work and enjoy just and favorable conditions at work, the right to privacy and confidentiality, and the right to freedom of expression and association. This has resulted in some economic, political, and social problems and, most importantly, psychological (mental health) problems. Some of these problems, such as the social and psychological problems, lead directly to the challenge of human interaction. Thus, we argue that the COVID-19 pandemic has led to human interaction challenges with some psychological (mental health) effects.

This pandemic is not just a human tragedy that is associated with the loss of millions of lives; it has also led to the harsh reality that humans must change their way of living to preserve and protect human life. At its heart is the right of association and all forms of close interactions that this right portends. Through self-isolation, physical distancing, the avoidance of handshakes, embraces, and other forms of expression of emotions, expressions of love and support, lockdown, and shutdown, humans are prevented from having physical interactions. Interaction is a crucial part of human social nature. According to Aristotle, humans are social beings [31]. As social beings, humans always and naturally yearn to interact with other humans. With the present situation, which is the result of the pandemic measures, humans have been forced to become introverted and withdrawn into themselves, instead of being extroverted and relational. This, in turn, has led to isolation and has increased the chances of mental health problems, such as depression, suicide, and emotional crisis. These emotional problems cause serious psychological conditions, due to the absence of human warmth, especially for those who have lost family members and loved ones to the pandemic. In addition, a lack of interaction can lead to the destruction of social cohesion and integration and can render humans as being ego solus and individualistic. In the words of Howard Phillips, “the mantra of social cohesion has been superseded by social distancing” [32]. Vasu Reddy elaborates this as he contends that

the social dimensions of the virus open up an archive of insights related to the physical sense of our experience as social beings. On a personal level, self-isolation—a dominant theme in the current language—is not merely about social distancing as a public health measure. A shutdown, with lockdowns and shelter-at-home orders, closed borders, and blocked airspace, implies a form of self-imposed exile. These tactics highlight the social impacts, and, by forcing us to physically distance ourselves from each other, will have implications for our location and relationship with others in the world [33].

What Reddy implies in the above quotation is that some of the pandemic measures can pose a serious psychological challenge to humans as a result of the limitation or absence of social interaction. Lack of social interaction is also affecting the family’s inherently social nature. The structure of family social life is rapidly collapsing as family members are forced to isolate themselves from each other when infected by the coronavirus. Those in lockdown in another locality are shut away from their family members and they are denied the privilege of having physical contact with them. Additionally, communal or social cohesion and relationships are gradually crumbling as families and friends who are infected within a given community are treated as outcasts in some places, making it difficult for people to relate to them or to care for them. Individuals are denied the right to freely travel and attend the ceremonies of their family members, loved ones, friends, and community members. In many countries, individuals are not allowed to attend the burials of their loved ones, regardless of the type of bond or relationship they share. With this development, communal or collective care and grief, complementarity, and the relationships that characterize human society are eroded.

The challenge of interaction, as related to the denial of the right to freedom of association, has an adverse impact not just on a person’s psychological well-being but also on their economic life. In this scenario, it becomes difficult, if not impossible, for workers to exhibit and realize their rights to collective bargaining. The International Labor Organization captures this vividly: “The COVID-19 crisis is, in many places, making the realization of rights to freedom of association and collective bargaining more difficult, both in law and practice, which, in turn, hampers the development of responses to the crisis rooted in social consensus” [34]. This is having a telling effect on workers’ economic life as many workers are retrenched from their paid job and some have their means of livelihood shut down due to the COVID-19 regulations and restrictions, such as lockdown and shutdown. This is because workers and other business owners are denied their right and opportunity “to exert agency or collective voice, to defend their rights and interests, or to positively influence the conditions of their working lives” [34] and achieve the smooth running of their businesses. The point here is that social dialog and interaction and the effective participation of socio-economic partners and unions are not allowed in many countries, resulting in the lack of a collective and representative voice. With this dimension, there is the increasing challenge of the loss of jobs and a high level of unemployment, and diminishing income. This readily leads to untold hardships, pain, suffering, and even death. Still, besides what is immediately obvious, the often-neglected impacts that are by far more devastating include loneliness, depression, trauma, suicidal thoughts, and general emotional breakdown.

The point we seek to make is that in this COVID-19 situation, there is a connection between the economic and the social spheres. As some have argued, “there is the consolidation of labor displacement, a growth in unemployment, and a deepening of inequality” [29,35]. Given that the means of livelihood of the masses are affected, their health is also in crisis. This is because the economy of the people affects their health. In many countries, those who are financially handicapped are denied basic and necessary medical care during this public health crisis. Another side to the health implications of the COVID-19 pandemic is that many lives are lost because some unguarded media communications in many nations have made it appear to be a death sentence for anyone infected, thus increasing the emotional stress on the part of those who worry about the disease on a daily basis. Therefore, with self-isolation and the practice of social distancing, which results in the denial of social interaction, many infected persons have no access to medical care as medics are also afraid of interaction. This is, arguably, the leading cause of death during this pandemic.

Our argument so far is that “the social life of COVID-19 is arresting, in as much as it is assaulting” [33] as it affects human social, economic, and health realities. The effect of this pandemic is at both the local and global levels [29]. This has challenged political leaders at both the national and global levels to think of a way forward. We want to argue that the pandemic should be confronted from the political, economic, and social angles, but should specifically focus on the social angle, as individuals’ mental health is affected. Unfortunately, the crisis conditions of the pandemic have become an opportunity for extreme individualism to rise in many places. In parts of Africa, such as South Africa, Nigeria, and Malawi, we learn of the massive embezzlement of funds allocated for the provision of palliatives and vaccines for the public. This is in addition to the challenge of human interaction (leading to mental health issues) posed by the pandemic itself. The question begging for an answer is: how can Afro-communitarianism serve as a viable tool for addressing the challenges of human interaction that create several mental health issues? This will be the focus of the next section.

### 1.3. Combating the Challenge of Human Interaction Posed by the COVID-19 Pandemic: A Proposal for Digital Communitarianism

Our argument is that the values of Afro-communitarianism can be employed to address the challenge of human interaction amid the COVID-19 pandemic. This will be done from two perspectives—methodological and axiological. From the methodological perspective, Afro-communitarianism explains the nature and importance of human relationships in society. Humans do not exist in isolation; they exist in a community. In these challenging times of the COVID-19 pandemic, where people are forced to self-isolate, humans are made to withdraw from the community and exist alone, leading to diverse mental health issues, such as clinical depression, suicide, emotional breakdown, etc. This is forcing humans to exist as individuals rather than as a community. The idea of humans living in isolation gives the impression that humans can exist alone and be independent of each other. This could somehow lead to individuals losing their identity. According to Metz, an individual’s identity is derived from the community in which that individual lives [23]. The practice of isolation (self-isolation) that the various governments of different countries have enforced takes individuals away from their community life and constrains them into private spaces where they can never realize their true selves. Afro-communitarianism maintains that it is in the community that humans can derive their identity. This argument stems from the social nature of humans as a species of animals that are always yearning for interaction. Afro-communitarianism emphasizes the need for individuals to coexist in the community. Humans can realize their being and who they are, not in isolation but in a group. The community or group gives them their identity. Thus, although the problem posed by the COVID-19 pandemic is serious, the attendant policies drafted to curb its spread ought not to be implemented in ways that encourage individualism by taking people away from their communities. Instead, these policies should and can be implemented in ways that pull individuals together within their communities.

Unfortunately, the implementation of the pandemic measures has confined humans to a pitiable situation. Using an Akan maxim, Gyekye buttresses this point as he asserts that “one, when a person descends from heaven, he (or she) descends into a human (or, human habitation) and two, solitariness (literally, walking alone) is a pitiable condition” [36]. Explaining the maxim, Olatunji Oyeshile contends that “a solitary individual, no matter his wealth, would lose the opportunity of benefiting from the helpfulness and co-operation that is abundant in the communal life” [37]. In contrast, Afro-communitarianism encourages fellow feeling, solidarity, and selflessness [1], which individualism abhors.

According to Menkiti [6,38] and Gyekye [15,36], a person realizes his/her being through the community. This is possible through his/her actions in the community, which must be moral. It is in this context that the axiological dimension of Afro-communitarianism comes to play. Afro-communitarianism not only gives a person his/her identity but also allows the person to be moral and to act morally. One can only become moral within a community. This is when he/she makes morally right decisions and acts morally in his/her dealings in society. The idea of self-isolation, lockdown, shutdown, etc., prevents individuals from existing in the community and acting morally in the community. If individuals are denied the opportunity to participate in the community’s daily life, they cannot lead a life that can promote communion, vitality, and the common good, which the ethics of Afro-communitarianism projects.

Given that individuals are not allowed to physically associate and socialize during this COVID-19 pandemic, they are also kept away from communing with one another. This act keeps people from quickly recognizing the economic and social challenges facing specific individuals in their localities. In this way, they fail to live in solidarity with one another. However, Afro-communitarianism provides the individual with the platform to define their identity and exhibit solidarity. Solidarity can only be expressed and exhibited within the community wherein the person finds him/herself. This community defines the individual and how he/she exhibits his/her communally oriented moral value and wealth [15].

The challenge that comes with the traditional view of Afro-communitarianism has to do with the social reality of self-isolation, physical distancing, lockdown, and shutdown, which prevent human physical interaction. Indeed, the traditional way in which Afro-communitarianism has been conceptualized, in terms of human physical interaction in the community, to enhance identity and solidarity cannot perfectly address the social reality of the COVID-19 pandemic. However, while self-isolation, physical distancing, lockdown, and shutdown challenge the realization of human physical interactions advocated in traditional Afro-communitarianism, all roads are not closed. There is an opening that we can tap into. This has to do with another social reality—electronic social media.

Through electronic social media, such as Facebook, Skype, WhatsApp, Zoom, etc., individuals can still interact. According to Aribiah Attoe and Jonathan Chimakonam, amid the COVID-19 pandemic and its restrictions on human physical interaction, these electronic social media become new ways in which humans can discover meaning in life. They also add that these media are opening new paths through which individuals exhibit self-sacrifice/care, solidarity, etc. [39]. With this new reality, there is the need to adapt Afro-communitarianism as an African culture-inspired social ideology that promotes human co-existence, complementarity, and solidarity via electronic social media, such as Facebook, Skype, WhatsApp, Zoom, etc. What is suggested here goes beyond the one-way traffic of information dissemination about the pandemic from governments and institutions to individual persons. We are talking about group- and community-based interactive approaches to issues concerning the well-being of individuals, not merely about the pandemic. Social media can be reconstructed and deployed as digital communities wherein people do those things that they do in normal times. Those who are in business can order their merchandise online and have their goods delivered to them. They, in turn, can open digital stores where they receive orders and deliver the goods to their customers. The same practice can be followed in various spheres of life. It is not as if this measure is not taking place already; it is. The problem is the limitations in scope and legal restrictions. For example, most small businesses, such as restaurants, were severely affected as a result of the forced lockdown. Our argument is that most businesses, especially SMEs, can be allowed to restart. Governments that are in association with tech companies can set up online marketplaces where transactions can continue to take place. Therefore, the restaurant owner and the employees, for example, can go to the restaurant, prepare meals, and deliver them to various homes while maintaining pandemic safety measures. Neighborhoods, streets, and local communities can set up group accounts where everyone can continue to interact in the same way as in a normal social community. We are talking of an informal system that can approximate the idea of a regular community digitally, but that can be more expansive to bring together people from different places and diverse groups.

There are two forms of digital communities that we can identify: formal and informal. With both forms, there is more global interaction, the realization of individual identity, and the practices of complementarity and solidarity. In this new context of Afro-communitarianism, individuals communicating via electronic social media can interact with people across the globe as a community, know and share their life challenges, provide counselling, support, and encouragement, and meet one another’s economic, health, and social needs as much as they can.

For the formal types of digital communities, social interaction via electronic media can help individuals to realize and exhibit their freedom of association denied them by the COVID-19 pandemic. With this, human beings in different places can organize their meetings and hold their events. For business and corporate establishments, where the staff mainly work from home, they can also organize their board meetings, union meetings, association meetings, conferences, workshops, etc., and engage in discussions and achieve collective bargaining, which has always been vital to arriving at consensus-based responses to corporate and business challenges or the crises of the unions. Through these avenues, there are bound to be “Negotiated agreements between employers’ and workers’ organizations” [34] and “collective agreement between workers and business owners on one side and the Government and employers of labor on the other side” [34]. Corporate management can continue to thrive. This type of formal interaction is possible and is currently happening on a large scale all over the world, through the relevant social media platforms, such as Zoom, Facebook, WhatsApp, etc.

However, the same cannot be said of the informal type. This is where the focus of our argument lies in this essay. By the “informal” type of digital communities, we are referring to normal social events and exchanges that occur in society. Granted that some societies, especially in the Western world, are quite individualistic, the need for a closer bond has never been as high as it currently is. In pre-pandemic times, people in such societies mainly located their human interactions at workplaces, entertainment facilities, and other indoor and outdoor events where they found some entertainment and the sharing of human warmth, most of the time with strangers. The pandemic, which has brought numerous emotional difficulties, has also highlighted the yearning for closer, more emotional relationships with friends and family. Many people all over the world are experiencing serious emotional cracks and other mental health issues, and require the sort of solidarity, caring, and sharing that one finds in Afro-communitarian settings. Our argument is to find ways of globally extending this sort of mutuality. It is easy enough to encourage family members or friends to set up such Afro-communitarian responses to the challenges of interaction posed by the pandemic, but in most cases, those who have more to give might not be family members or friends. However, many people who are deeply affected by the pandemic have neither family nor friends. Additionally, setting up larger communities promises the availability of deeper and broader support resources for people going through various levels of emotional and psychological difficulties. The informal types of digital communities are, thus, not the sort that can be well-supported by the current digital tools available such as Zoom, WhatsApp, Facebook, Twitter, Instagram, Skype, etc. In these tools, the focus is usually entertainment, and the members of such communities remain strangers who might not be keen to share, care, and engage in a relationship of solidarity with others. For these informal digital communities to materialize, new tools are required. Governments and institutions around the world need to partner with technology companies to set up such media tools.

Thus, we argue that amid the COVID-19 pandemic, Afro-communitarianism can function as an ideology that can help us to redefine our course of existence as humans. Given that Afro-communitarianism advocates solidarity, we ought to go beyond the limitations of self-isolation, lockdown, and shutdown to find ways to connect individuals in the community, where social media can help us to identify people’s problems and come to their aid. In these times, we ought to exhibit the values of “sharing resources, burdens, and social responsibility, mutual aid, caring for others, interdependence, solidarity, reciprocal obligation, social harmony, and mutual trust” [15]. The practice of these values will help make society better, despite the scourging effect of the COVID-19 pandemic. This is to say that Afro-communitarianism leads to selfless and sacrificial service toward others, compared to individualism, which leads to the kind of self-centered and selfish behavior that is pivotal to embezzlement and the misuse of funds meant for the fight against the effect of the COVID-19 pandemic.

Our argument is that Afro-communitarianism places the burden of solidarity on the individuals instead of on the community. The various governments ought to make the society conducive for their citizens, given that a moral society humanizes and moralizes its citizen. The governments of various countries need to implement measures that will involve the citizens in the fight against some of the neglected challenges posed by the pandemic and its measures. Our argument is that both society and individuals ought to be morally sensitive by showing care and concern for others in need, especially in these challenging COVID-19 times.

One could ask, then, how can the medical world be incorporated into our idea of a digital Afro-communitarian framework in the fight against the COVID-19 pandemic? As we have stated earlier, the COVID-19 pandemic has posed a challenge in terms of social interaction, which also has medical implications, such as the emotional and psychological breakdown that has resulted in some untimely deaths due to a lack of adequate medical care. Hence, our thought here is that the governments of various countries of the world should partner with technology companies to build a digital medical community that will bring both medical practitioners and patients together so that they can interact on medical issues. In this way, those with medical conditions and illnesses can relate their medical challenges to medical practitioners, who will in turn provide them with possible and readily available medical solutions, advice, and counselling, based on their needs. This digital medical community, it must be stated, will bring medical services to the doors of those in need of medical aid, while seeing to it that the medical personnel and practitioners are safe and sound. Additionally, through this digital social community, more severe medical challenges and conditions requiring physical medical attention could be scheduled and addressed accordingly. With this possibility, human interaction can be maintained, while the spread of the COVID-19 virus is curtailed.

It could also be asked, what is the essence of this article? Our point, so far, is that social interaction and, in this era of the COVID-19 pandemic, digital social interaction, when informed by the values of Afro-communitarianism, can help humans ameliorate most of the mental health, medical, and social challenges with which the pandemic confronts humanity. Thus, our position in this article is that digital Afro-communitarianism is a viable tool for addressing the mental effects of the COVID-19 pandemic because it encourages humans to think about the collective good, wherein everyone’s need in human society is the concern of all. This is to say that digital Afro-communitarianism advocates that communal values such as care, complementarity, hospitality, and solidarity should be practiced by all and sundry during the COVID-19 pandemic, but that this should take place within a digital space. Hence, the presence of the COVID-19 virus and pandemic must not be an excuse to relegate valuable human social interaction into the background and thereby exacerbate mental health issues. Instead, we have demonstrated that humans can still be involved in social interaction but at the digital level during this pandemic, in order to practice the values of care, complementarity, hospitality, and solidarity, which will, in turn, promote communion (harmony (harmonious coexistence)), vitality, and the common good.

## 2. Conclusions

In this paper, we have argued that Afro-communitarianism can help us to address the challenge of human interaction, the absence of which can cause several mental health issues during this COVID-19 pandemic. We have acknowledged that the COVID-19 pandemic and the measures to prevent its further spread, such as self-isolation, lockdown, shutdown, etc., while being important, deny humans their fundamental human rights, dehumanize them, and make them individualistic, thereby causing all sorts of mental and emotional problems. We argue that Afro-communitarianism, which promotes communion, vitality (life), sharing, caring, solidarity with others, and the common good, can help us to cope with some of the social challenges of the COVID-19 pandemic through the formation of relevant digital communities that are adequately informal and collective.

Although we have argued that digital Afro-communitarianism can function as a viable tool for addressing the challenges of social interaction posed by the COVID-19 pandemic, it is germane to note that digital Afro-communitarianism has implications for other human existential/phenomenological challenges beyond mere social interaction. Thus, the task for future research is for researchers and scholars to seek out other human existential/phenomenological challenges and then employ digital Afro-communitarianism to address them. In addition, while we have employed the methodological and axiological dimensions of Afro-communitarianism to address the challenge of social interaction during the COVID-19 pandemic, other researchers/scholars can employ other dimensions of Afro-communitarianism to address other human existential/phenomenological challenges. The research into Afro-communitarianism and, specifically, digital Afro-communitarianism continues.

## Data Availability

Not applicable.
